# Supplementary material for: Intravital mesoscopic fluorescence molecular tomography allows non-invasive in vivo monitoring and quantification of breast cancer growth dynamics
Source: Commun Biol. 2021 May 11;4:556. doi: 10.1038/s42003-021-02063-8 (PMC8113483; doi:10.1038/s42003-021-02063-8)
Supplement: Supplementary file 1 — Supplementary Information [file 42003_2021_2063_MOESM1_ESM.pdf]

Supplementary Information:

Intravital mesoscopic fluorescence molecular tomography allows non-invasive in vivo monitoring and quantification of breast cancer growth dynamics

Mehmet S. Ozturk[1,2], Marta G. Montero[1], Ling Wang[1], Lucas M. Chaible[1], Martin Jechlinger[1,3,*], and Robert Prevedel[1,*]

[1] Cell Biology and Biophysics Unit, European Molecular Biology Laboratory (EMBL), Heidelberg, Germany

[2] Present address: Electrical and Electronics Engineering Department, Karadeniz Technical University, Trabzon, Turkey

[3] Present address: MOLIT Institut gGmbH, Heilbronn, Germany

[*] Correspondence should be addressed to: martin.jechlinger@molit.eu or prevedel@embl.de

Supplementary Note 1: Double-integrating-sphere setup for measuring bulk optical properties of mouse skin tissue

To measure bulk optical properties of mouse tissue, we utilized a double-integrating sphere setup (DIS) with the sample in the middle [1,2] (**Supplementary Figure 1**). From transmittance, diffuse and reflected light measurements the optical properties can be estimated using the inverse adding doubling (IAD) method proposed by Prahl et. al. [3,4]**.** Each integrating sphere (EverFine Corporation, Hangzhou, China) was equipped with a ϕ=3.6 mm Si photodiode (PDA36A2, Thorlabs Inc., New Jersey, USA) whose voltage readings were digitized by a 16-bit DAQ card (PCIe-6323, National Instruments, Texas, US). The inner walls of the integrating spheres were coated with barium sulfate and had a diameter of 30 cm. A supercontinuum laser (SC-Pro-7, YSL Photonics, Wuhan, China) in combination with an acousto-optic tunable filter (AOTF) device (AOTFnC-VIS, AA Opto-Electronics, Orsay, France) provided a rapid wavelength scanning light source over the band of 550 – 720 nm. The power spectra (**Supplementary Figure 1** inset) at the fiber exit port were calibrated by combining a spectrometer (USB4000, Ocean Insight, US) and a powermeter (PM100D, Thorlabs Inc., New Jersey, USA). Immediately after the AOTF, the monochromatic light beam was coupled into a 50 μm core diameter multi-mode fiber. A collimator lens with f = 11 mm generated a round light spot (ϕ ~ 3 mm) on the sample positioned between the spheres. Detector ports were masked from the sample and entrance ports by internal baffles, in order to ensure the detection of diffuse light only. Because of the sphere geometry, the sample is illuminated under an angle of 9°. Both diffuse and reflected (including specular reflectance) light were measured simulataneously by the detectors. We used a custom-designed and 3D-printed sample holder made from black polyether ether ketone (PEEK) which fit the sphere ports precisely and had a ϕ = 10 mm access hole. Before measurements, the light beam was aligned to be focused at the center of the access hole. The skin sample was taken from the mammary gland area of the sacrificed mouse (following FELASA guidelines). First, the mouse skin was

separated from the peritoneum, avoiding any tissue shrinkage and then was sandwiched by two microscope glass slides with thickness of 1.00 mm. Once a sample was inserted into the sample holder and positioned between the spheres, measurements were performed by home-made LabView control program which synchronized the digitalization to the wavelength scanning. Data for each wavelength were averaged from 1,000 consecutive measurements with a 10kHz sampling rate. An entire measurement over the whole wavelength range could be finished within 1 minute. The measurements along with the calibration data were preprocessed followed by the optical property estimation using the IAD approach. **Supplementary Figure 2** (a) and (b) show the so measured scattering and absorption coefficients of two mouse skin samples, respectively.

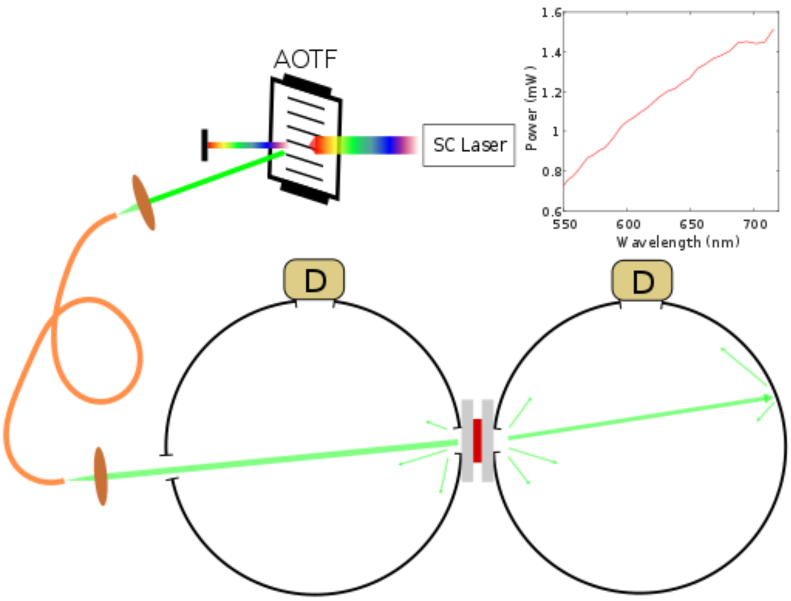

Supplementary Figure 1: Schematic overview of the double integrating sphere system in the 550-720 nm range. Inset: Light power spectrum at the fiber exit port when the SC laser functions at the maximum output. SC laser = Supercontinuum laser; D = photodiode detector; AOTF = Acousto-optic tunable filter.

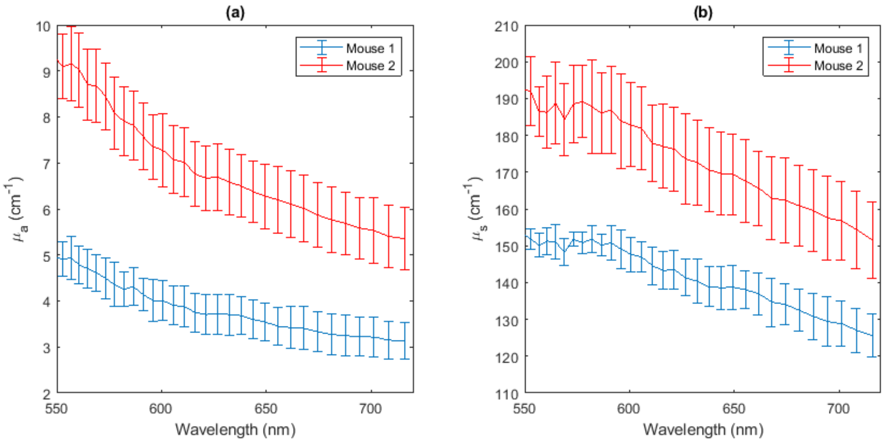

Supplementary Figure 2: Optical properties of mouse skin. (a) Absorption coefficient and (b) scattering coefficient in the 550-720 nm range. Data are averaged from 3 measurements of different locations and the error bars denote standard deviation. The skin thicknesses of mouse 1 and 2 were 0.55 mm and 0.50 mm, respectively, as measured by a caliper. The anisotropy factor (g) and refractive index of skin were assumed as 0.9 and 1.4, respectively [5].

Supplementary Note 2: OCT imaging of mouse skin

The performance of the NIVOW was evaluated by a self-built spectral-domain Optical Coherence Tomography (SD-OCT) setup with a linear wavenumber space spectrometer, whose design followed Supplementary reference [6]. Briefly, the spectrometer of the SD-OCT setup utilized a 28 kHz line camera (AViiVA SM2 CL, e2v, Cedex, France) covering a wavenumber range of 8.267–6.830 μm^{-1} (center wavelength 832 nm). The measured axial resolution in tissue (n=1.35) was ~3.5 μm. The transversal light beam scanning was achieved by a combination of galvo mirror pair and a telecentric scanning lens (f= 60.3 mm). When the laser beam diameter was 4.0 mm, the transversal resolution at the focal plane was measured to be 23.8 μm. Intensity profile of each depth scan (A-line) was reconstructed from interference spectral signal following a regular OCT postprocessing procedure including steps of dispersion compensation, background subtraction, spectrum reshaping and inverse fast Fourier transformation [7]. When the line camera speed was 28 kHz, a cross-sectional-sectional image (B-scan) containing 128 A-lines of 2048 depth samples took ~4.57 ms to acquire. Consequently, the time-lapse images were acquired with a rate of 54.8 frames/s. The transversal spatial scale of the OCT imaging system was calibrated by placing an array camera (CM3-U3-50S5M-CS, FLIR, Wilsonville, USA) at the focal spot while the axial scale was calibrated by a 1.00 mm thickness air gap between two regular microscope slides. In order to quantify the tissue movement during the experiment, a cross section of the mammary gland tissue area was imaged by the OCT at full speed (**Supplementary Movie 1 & 2**). Every consecutive image pair of these image series was digitally registered with a resolution of 1/10 pixel followed by pixel-micrometer conversion with the calibrated spatial scale factors in both axial and transversal directions [8,9]. The spatial shifts of each frame relative to the first were then plotted in **Fig. 1e**.

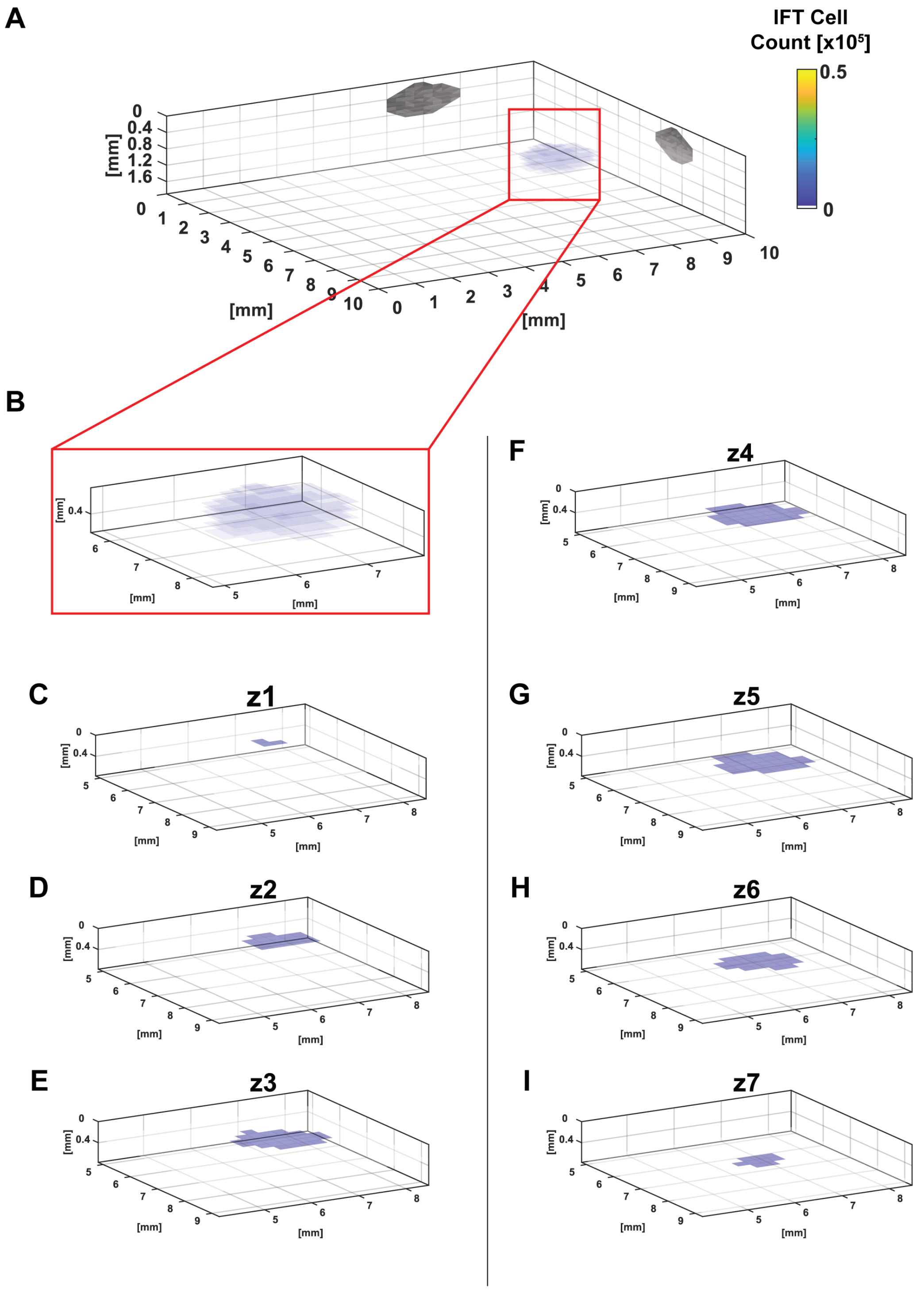

Supplementary Figure 3 Slice-by-slice visualization of IFT 3D tumor reconstruction. A. An IFT tumor reconstruction in 3D is shown. The panel also shows the projections (i.e. shadows) on two axial plane x-z and y-z. B. A close-up image of the reconstruction is shown. C-I. Each layer (slice) of the reconstruction is shown individually, which are not directly visible in A./B. due to the transparancy and overlapping of all layers. Color bars represents the IFT cell count per voxel.

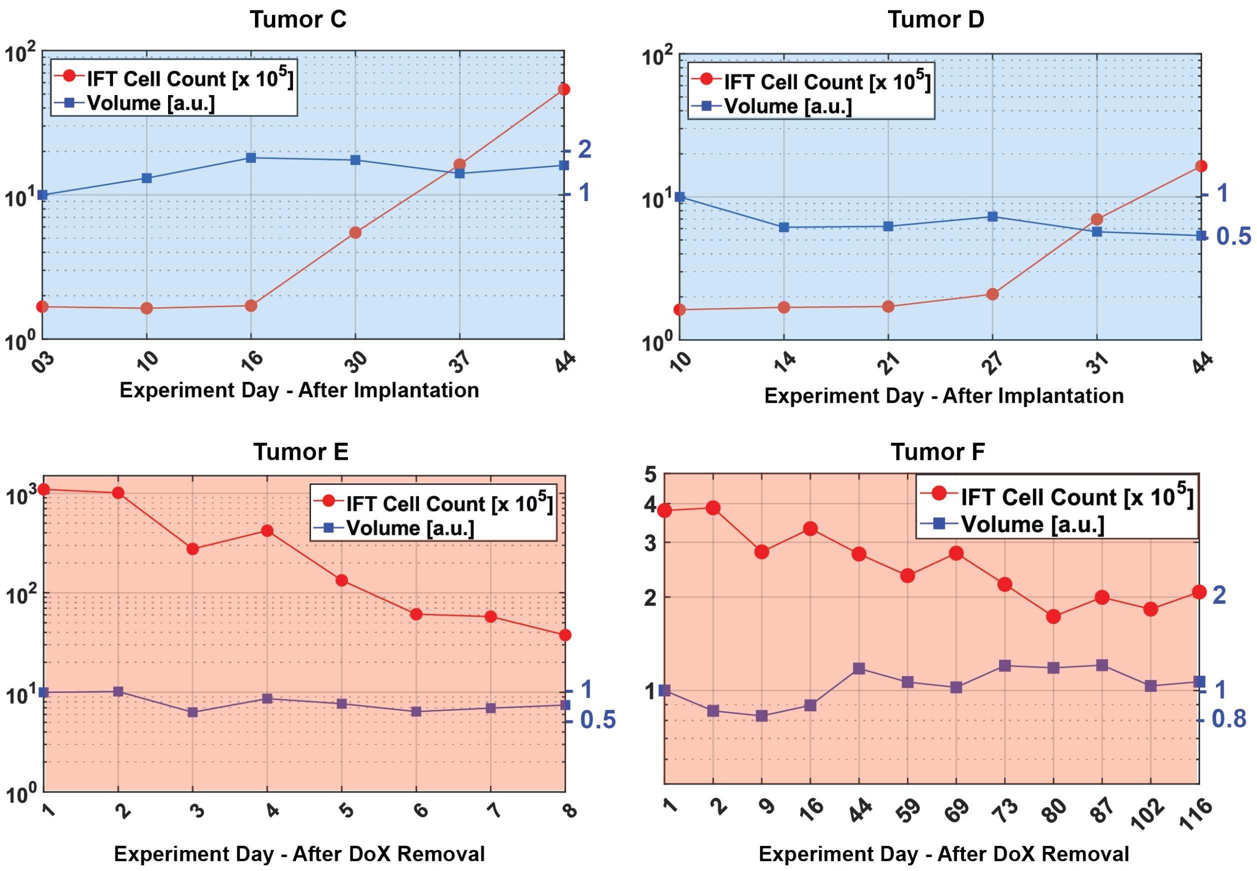

Supplementary Figure 4 Two tumor progression and two tumor regression scenarios reliably detect changes in tumor cell count over time. Quantitative cell count (red) and relative volume change over time (blue) normalized to the first day of imaging for Tumor C, D, E and F is shown. Tumor C and D shows the progression from two different animals during 44 days while both animals were on doxycycline diet. C-D. Tumor E and F shows the regression of tumors after the retrieval of doxycycline from two additional animals.

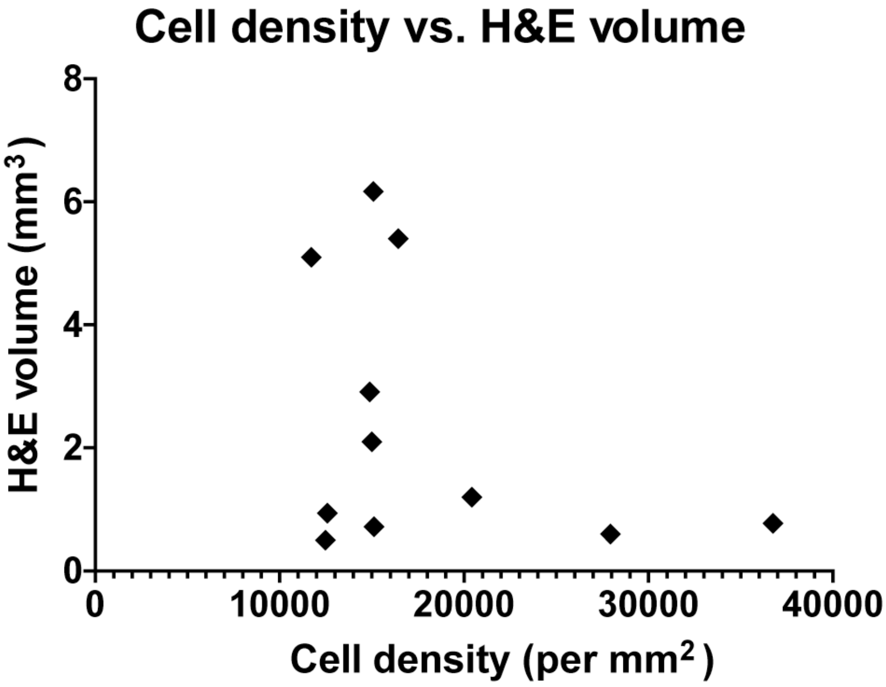

Supplementary Figure 5 H&E volume vs. cell density (as defined as number of tumor cells per mm^2). H&E volume was reconstructed from individual H&E slices (on average 50µm distance between each slice) throughout each tumor (n=11). Cell density of tumor cells was calculated using our sub-area analysis approach as described in Fig. 3 and main text. This shows that the cell density of similarly sized tumors can vary significantly due to the different amounts of non-fluorescent, stromal cells that infiltrate the tumors. Thus, there is no correlation between the tumor cell density and volume of tumors.

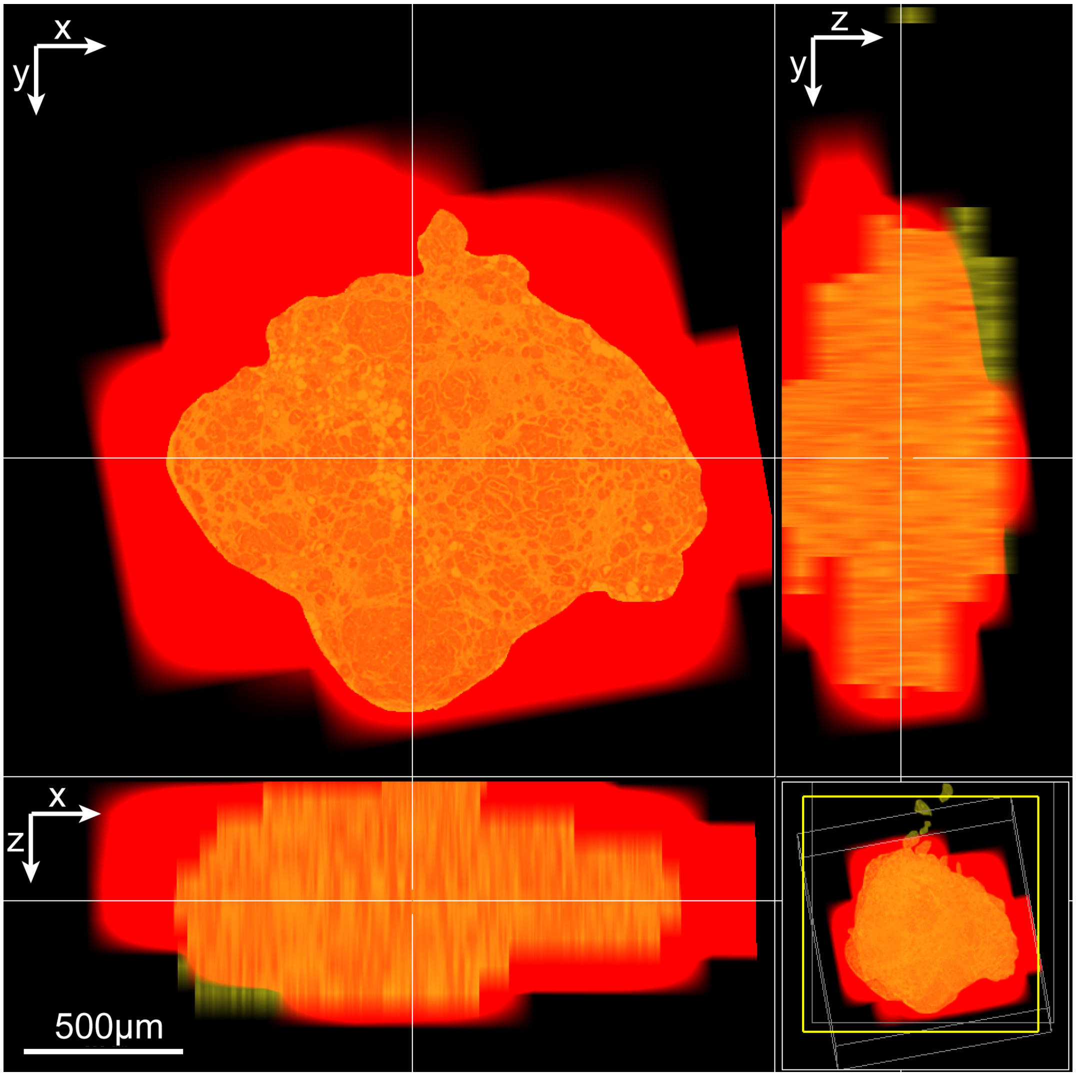

Supplementary Figure 6 Visual overlay of tumor volume reconstructions based on H&E (yellow) and IFT (red). The H&E tumor volume was measured to be 0.68mm^3 – here, shrinkage during the H&E fixation and staining process (by ~37% in volume, see Ref. [15]) has been corrected for. The IFT reconstruction quantitatively matches the H&E volume outline and yields a volume of ~0.9mm^3 . The residual discrepancy mainly stems from the relatively large pixel size of IFT (250x250x100 µm) which overestimates the IFT volume by at least half the resolution on the border voxels. If the uncertainty due to the resolution of IFT is also accounted for, then the volume estimation becomes 0.71mm^3, leaving only 4% difference against the ground truth.

H&E slice depth [um]	Cell density (per mm^2)	Cell count based on the cell density	Cell count based on the min. cell density	Cell count based on the max. cell density
72	11538	9591	8332	13195
108	11821	31052	26330	41697
144	15873	94660	59774	94662
180	10023	75434	75433	119460
216	12598	119783	95301	150924
252	12761	127484	100134	158578
315	11653	105931	91114	144294
387	14668	102229	69855	110627
423	14477	83088	57523	91097
459	10868	46440	42830	67828
495	11886	21066	17765	28133
531	11854	1470	1243	1968
567	13636	1236	908	1439
**Total 3D cell count**		819463	646543	1023902

Min. error (%)	Max. error (%)
-21.10	24.95

Supplementary Table 1 Error analysis of our sub-area cell counting approach on a representative tumor. To estimate the cell counting variability, we compared our approach based on slice-by-slice sub-area analysis to the extrapolation obtained with either the overall minimum (blue) or maximum (grey) cell density found across all slices. This yields approximate lower and upper boundaries of the expected error, in this case <25%.

Supplementary Note 3: IFT reconstruction thresholding workflow

Below we describe our thresholding workflow which combines different aspects of standard practices from Near-Infrared spectroscopy[10] and medical image processing[11,12]. This allowed us to minimize the user input while thresholding the IFT reconstruction in an unbiased manner.

The following steps were taken for our mutual information-based threshold selection:

1. The raw data is thresholded based on the median value of the entire data set, i.e. signal below the median is set to zero. This serves to remove any system-based noise.
2. A 3D reconstruction is performed which yields the 'raw' reconstruction (without any thresholding). This yields a 3D data set as depicted in **Supplementary Supplementary Figure7** below.

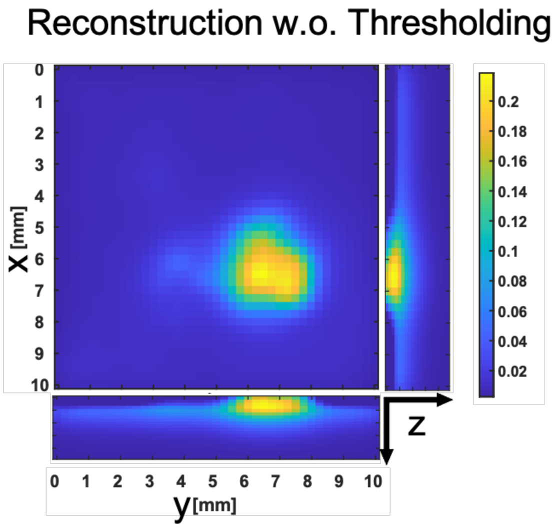

Supplementary Figure 7: A representative output of the reconstruction algorithm is depicted. No thresholding is applied on the data. Highly confined tumor signal was reconstructed, the remaining data is under the noise level.

3. At this stage a threshold value for the 'raw reconstruction' needs to be chosen. For this, a so-called 'Center detector' image is retrieved from the raw data, which corresponds to zero source-detector separation and is thus equivalent to an epifluorescence microscopy image (i.e. diffuse reflectance image).
4. To mitigate the blurring effect due to the scattering, we threshold the center detector image between the maximum intensity and 2 standard deviation below the mean signal. We note that because of the inherent sample variability, this needs to be recalculated for each new data set[13]. This thresholded center detector image served as a 'reference image' for identifying the threshold value for the reconstruction (see **Supplementary Supplementary Figure 8**, left panel, below).

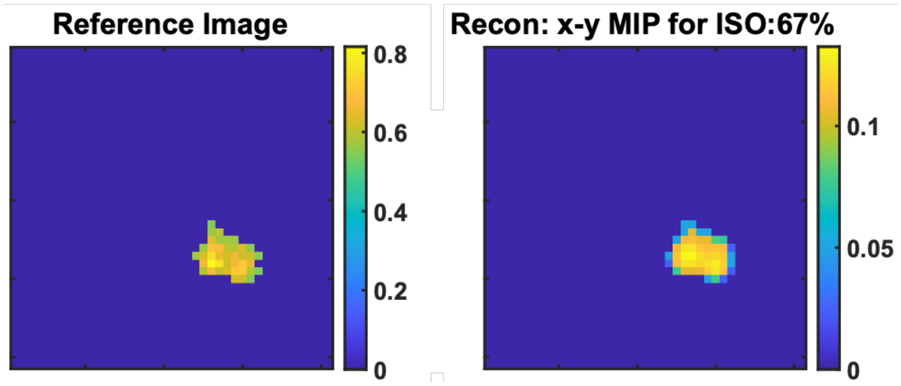

Supplementary Figure 8: Left: Reference image thresholded as described above, i.e. any signal between the maximum and 2 standard deviation below the mean was kept with the rest set to zero. Right: Best threshold (ISO) value identified during mutual information thresholding. In this particular case, reconstructed values below 67% of the maximum are discarded as opposed to the common practice of 50% in the field[14].

5. A maximum intensity projection (MIP) over the z-axis of the 'raw reconstruction' is performed, yielding the same sized image as the reference image (**Supplementary Supplementary Figure 88**, right panel, above).
6. The reference image and the MIP of the reconstruction are compared by the so-called mutual information metric, which is commonly used in the image processing community to conduct registration studies[11]. An iterative process was conducted by gradually increasing the threshold value (ISO value). For every updated ISO value, a mutual information metric was calculated between the reference image and the MIP of the reconstruction (**Supplementary Supplementary Figure 9**). **Supplementary Movie 4** provides a visual explanation of this iterative thresholding workflow.

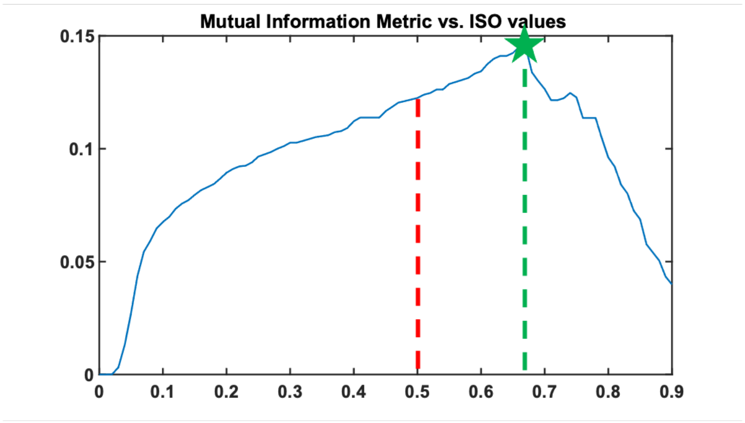

Supplementary Figure 9: The y-axis represents the mutual information (MI) value while the x-axis is the ISO (threshold) value. For each ISO iteration, an MI value is calculated and the global maximum corresponds to the highest similarity between the reference image and the MIP of the reconstruction (0.67, green dashed line in this case). For comparison, the common practice for ISO (threshold) value is also shown (0.50, red dashed line).

7. The threshold value for the maximum mutual information metric is chosen and used for 3D visualization (see **Supplementary Supplementary Figure**).

As can be seen in the direct comparison in Figure 3.4, the MI-based thresholding delivers approximately half the volume of the standard 50% percent thresholding (~1mm^3 vs. ~2mm^3, respectively), and is therefore much closer to the actual volume of 0.68mm^3 as assessed by H&E reconstruction (also see **Supplementary Figure 6).** This example showcases the discrepancy if the common practice of a 50% threshold would be adapted. Here we would like to note that for this example a confined tumor was chosen. In our empirical experience the difference between the 50% thresholding and our MI thresholding can be much higher in the case of more spatially spread-out tumors.

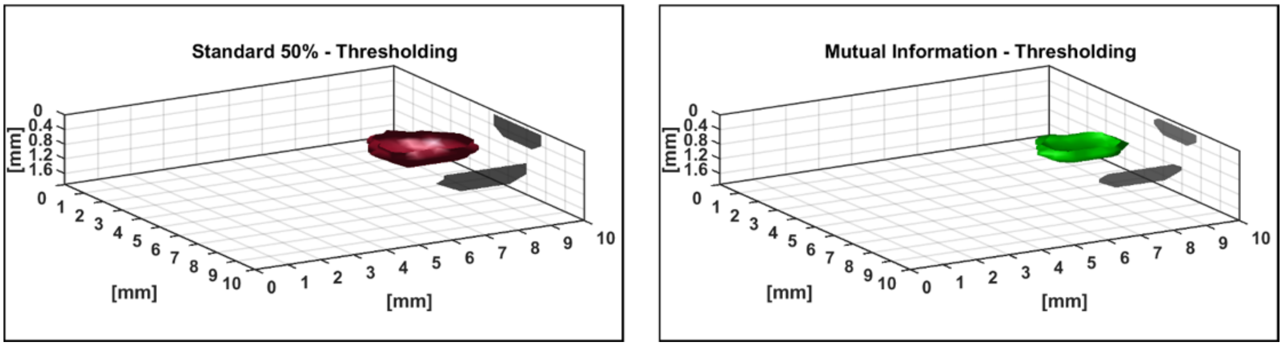

Supplementary Figure 10: Left: IFT reconstruction with 50% thresholding – total volume ~2mm^3. Right: Our optimal Mutual Information metric thresholding result corresponding to a 67% threshold – total volume ~1mm^3.

Supplementary References

1. Aernouts, B. *et al.* Supercontinuum laser based optical characterization of Intralipid® phantoms in the 500-2250 nm range. *Opt. Express* **21**, 32450 (2013).
2. Pickering, J. W. *et al.* Double-integrating-sphere system for measuring the optical properties of tissue. *Appl. Opt.* **32**, 399 (1993).
3. Prahl, S. A. Everything I think you should know about Inverse Adding-Doubling. 72 (2010).
4. Prahl, S. A., van Gemert, M. J. C. & Welch, A. J. Determining the optical properties of turbid media by using the adding–doubling method. *Appl. Opt.* **32**, 559 (1993).
5. Cheong, W.-F., Prahl, S. A. & Welch, A. J. A Review of the Optical Properties of Biological Tissues. *IEEE J. Quantum Electron.* **26**, 2166–2185 (1990).
6. Lan, G. & Li, G. Design of a k-space spectrometer for ultra-broad waveband spectral domain optical coherence tomography. *Sci. Rep.* **7**, 1–8 (2017).
7. Liba, O., Sorelle, E. D., Sen, D. & Zerda, A. De. Contrast-enhanced optical coherence tomography with picomolar sensitivity for functional in vivo imaging. *Sci. Rep.* **6**, 23337 (2016).
8. Guizar-Sicairos, M., Thurman, S. T. & Fienup, J. R. Efficient subpixel image registration algorithms. *Opt. Lett.* **33**, 156–8 (2008).
9. Wang, L. *et al.* Highly reproducible swept-source, dispersion-encoded full-range biometry and imaging of the mouse eye. *J. Biomed. Opt.* **15**, 046004 (2010).
10. Yücel, M. A. *et al.* Best Practices for fNIRS publications. *Neurophotonics* **(in revisi**, 1–34 (2020).
11. Yim, Y., Wakid, M., Kirmizibayrak, C., Bielamowicz, S. & Hahn, J. Registration of 3D CT Data to 2D

Endoscopic Image using a Gradient Mutual Information based Viewpoint Matching for Image-Guided Medialization Laryngoplasty. *J. Comput. Sci. Eng.* **4**, 368–387 (2010).

12. Liu, T. T., Nalci, A. & Falahpour, M. The global signal in fMRI: Nuisance or Information? *Neuroimage* **150**, 213–229 (2017).

13. Guven, M., Yazici, B., Intes, X. & Chance, B. Diffuse optical tomography with a priori anatomical information. *Phys. Med. Biol.* **50**, 2837–2858 (2005).

14. Zhao, L., Yang, H., Cong, W., Wang, G. & Intes, X. L_p regularization for early gate fluorescence molecular tomography. *Opt. Lett.* **39**, 4156 (2014).

15. Tran, T. *et al.* Correcting the Shrinkage Effects of Formalin Fixation and Tissue Processing for Renal Tumors: toward Standardization of Pathological Reporting of Tumor Size. *J. Cancer* **6**, 759–766 (2015).
