# Supplementary material for: Intravital mesoscopic fluorescence molecular tomography allows non-invasive in vivo monitoring and quantification of breast cancer growth dynamics
Source: Commun Biol. 2021 May 11;4:556. doi: 10.1038/s42003-021-02063-8 (PMC8113483; doi:10.1038/s42003-021-02063-8)
Supplement: Supplementary file 2 — Description of Additional Supplementary Files [file 42003_2021_2063_MOESM2_ESM.pdf]

Title: Supplementary Movie 1:
Description: Time-lapse cross-sectional images of mouse abdomen skin over 9.4 s obtained by the OCT setup without the NIVOW. Scale bar 100 μm.

Title: Supplementary Movie 2:
Description: Time-lapse cross-sectional images of mouse abdomen skin over 9.4 s obtained by the OCT setup with the NIVOW. Scale bar 100 μm.

Title: Supplementary Movie 3:
Description: Non-invasive, vacuum-operated stabilization window (NIVOW) in operation. This video shows the use of the NIVOW on a living mouse. Reduced tissue motion is apparent below the window.

Title: Supplementary Movie 4:
Description: Iterative thresholding workflow based on mutual information metric. This video shows the change in mutual information with increasing threshold value together with the thresholded MIP of the reconstruction as well as the reference image of an exemplary tumor. Furthermore, towards the end of the video the comparison to a 50% thresholded 3D reconstruction is shown.

Title: Supplementary Data 1:
Description: Mouse skin position as a function of time as measured by OCT, showing tissue motion along z and x axes without and with NIVOW.

Title: Supplementary Data 2:
Description: In-vitro control experiment of glass capillaries filled with fluorescein of different concentrations show a linear relationship in their reconstructed IFT signal.

Title: Supplementary Data 3:
Description: Integrated IFT reconstruction signal values for 8 tumors of varying size and intensity plotted against 'ground-truth' cell count as obtained by H&E analysis.

Title: Supplementary Data 4:
Description: Longitudinal IFT data of cell count and tumor volume for two different animals c.f. Fig.4a,b.
