# Supplementary material for: Intravital mesoscopic fluorescence molecular tomography allows non-invasive in vivo monitoring and quantification of breast cancer growth dynamics
Source: Commun Biol. 2021 May 11;4:556. doi: 10.1038/s42003-021-02063-8 (PMC8113483; doi:10.1038/s42003-021-02063-8)
Supplement: Supplementary file 11 — Reporting Summary [file 42003_2021_2063_MOESM11_ESM.pdf]

nature research

Corresponding author(s): Dr. Robert Prevedel
Dr. Martin Jechlinger

Last updated by author(s): Mar 15, 2021

Reporting Summary

Nature Research wishes to improve the reproducibility of the work that we publish. This form provides structure for consistency and transparency in reporting. For further information on Nature Research policies, see our Editorial Policies and the Editorial Policy Checklist.

Statistics

For all statistical analyses, confirm that the following items are present in the figure legend, table legend, main text, or Methods section.

n/a	Confirmed	
☐	☒	The exact sample size (n) for each experimental group/condition, given as a discrete number and unit of measurement
☐	☒	A statement on whether measurements were taken from distinct samples or whether the same sample was measured repeatedly
☒	☐	The statistical test(s) used AND whether they are one- or two-sided<br>*Only common tests should be described solely by name; describe more complex techniques in the Methods section.*
☒	☐	A description of all covariates tested
☒	☐	A description of any assumptions or corrections, such as tests of normality and adjustment for multiple comparisons
☒	☐	A full description of the statistical parameters including central tendency (e.g. means) or other basic estimates (e.g. regression coefficient) AND variation (e.g. standard deviation) or associated estimates of uncertainty (e.g. confidence intervals)
☒	☐	For null hypothesis testing, the test statistic (e.g. F, t, r) with confidence intervals, effect sizes, degrees of freedom and P value noted<br>*Give P values as exact values whenever suitable.*
☒	☐	For Bayesian analysis, information on the choice of priors and Markov chain Monte Carlo settings
☒	☐	For hierarchical and complex designs, identification of the appropriate level for tests and full reporting of outcomes
☒	☐	Estimates of effect sizes (e.g. Cohen's d, Pearson's r), indicating how they were calculated

Our web collection on statistics for biologists contains articles on many of the points above.

Software and code

Policy information about availability of computer code

Data collection	All software used to collect the data in this paper are described in the methods section and are available from the corresponding author on reasonable request. source.
Data analysis	All software used to analyze the data in this paper are described in the methods section and are available from the corresponding author on reasonable request.

For manuscripts utilizing custom algorithms or software that are central to the research but not yet described in published literature, software must be made available to editors and reviewers. We strongly encourage code deposition in a community repository (e.g. GitHub). See the Nature Research guidelines for submitting code & software for further information.

Data

Policy information about availability of data

All manuscripts must include a data availability statement. This statement should provide the following information, where applicable:
- Accession codes, unique identifiers, or web links for publicly available datasets
- A list of figures that have associated raw data
- A description of any restrictions on data availability

The datasets generated and software code utilized in the current study are available from the corresponding author on reasonable request.

Field-specific reporting

Please select the one below that is the best fit for your research. If you are not sure, read the appropriate sections before making your selection.

- [x] Life sciences
- [ ] Behavioural & social sciences
- [ ] Ecological, evolutionary & environmental sciences

For a reference copy of the document with all sections, see nature.com/documents/nr-reporting-summary-flat.pdf

Life sciences study design

All studies must disclose on these points even when the disclosure is negative.

Sample size	No prior assumptions were made regarding effect sizes. All relevant and possible data points were used for statistical comparisons when applicable.
Data exclusions	No acquired data was excluded from the analysis
Replication	Biological experiments were performed independently of each other, including media preparation.
Randomization	Primary mammary epithelial cells were harvested under the same conditions for all experiments; For experiments in vivo, animals were injected in the fat pad #4 of Rag1 -/- female mice with an optimized number of 150.000 cells in 40µl of media and induced to grow a tumor. Single animals were followed over time performing non-invasive longitudinal IFT. No randomization was applicable.
Blinding	In these experimental cases, blinding was not possible, due to the need to maintain specific experimental conditions for tumor induction/ regression.

Reporting for specific materials, systems and methods

We require information from authors about some types of materials, experimental systems and methods used in many studies. Here, indicate whether each material, system or method listed is relevant to your study. If you are not sure if a list item applies to your research, read the appropriate section before selecting a response.

Materials & experimental systems

n/a	Involved in the study	
☒	☐	Antibodies
☒	☐	Eukaryotic cell lines
☒	☐	Palaeontology and archaeology
☐	☒	Animals and other organisms
☒	☐	Human research participants
☒	☐	Clinical data
☒	☐	Dual use research of concern

Methods

n/a	Involved in the study	
☒	☐	ChIP-seq
☒	☐	Flow cytometry
☒	☐	MRI-based neuroimaging

Animals and other organisms

Policy information about studies involving animals; ARRIVE guidelines recommended for reporting animal research

Laboratory animals	A detailed statement regarding strain, background and prior literature describing the used mice can be found in Materials and Methods section submitted
Wild animals	The study did not involve wild animals
Field-collected samples	The study did not involve field-collected samples
Ethics oversight	Breeding and maintenance of mouse colony was done in LAR (Laboratory Animal Resources) facility of EMBL Heidelberg (IUCUC protocol MJ160070), in accordance to the guidelines of the European Commission, revised Directive 2010/63/EU and AVMA Guidelines 2007, under veterinarian supervision. All effort were made to use the minimal amount of animals as possible in accordance with Russell and Burch's principle of (3Rs) reduction and highest ethical standard.

Note that full information on the approval of the study protocol must also be provided in the manuscript.
